# Translation of the Mr. Stromeyer's Letter Concerning the Vaccine Innoculation Practiced by Him

**Published:** 1800-05

**Authors:** 


*The following Letter was communicated by Mr.* HUNNEHMAN, *of Mortimer Street, April* 18, 1800.

---

Tranflation of Mr. STROMEYER's Letter concerning the Vaccine Inoculation practifed by him.

*Hanover, March* 24, 1800.

THIS year we have inoculated forty perfons, as well with the vaccine matter received of Dr. Pearfon, as that of Dr. Jenner, all of which went properly through the difeafe.

 Betwixt

Betwixt the London and Gloucester vaccine matter, it appears to me, there subsists an essential difference. The London matter produces frequently an eruption of small pimples, but they disappear within a day or two at furthest. Dr. Pearson calls these eruptions pimples. The Gloucester matter has never produced this effect here; but it frequently *occasioned ulcerations of the inoculated part, of a tedious and long duration, which the former matter never did*; on account of which I now only make use of Dr. Pearson's vaccine matter.

The nettle-fever like eruptions I have observed several times, but *never that sort of eruption, repeatedly noticed in London, which so much resembles the small-pox.*

Of all those I have inoculated, none of them were severely ill during the disease; their general symptoms were, a slight fever, depression of spirits, and a pale countenance. A few instances, in which the inoculated part arrived to a due degree of perfection, and where a swelling of the axillary glands was observed, no signs of any illness were present, at least no symptoms could be noticed, which proved the infection to have acted upon the whole system. These latter ones I shall re-inoculate; and in case the result should be the same as the first, would it be fair to suppose them safe against the small-pox infection?

The most of our physicians here exclaim against the vaccine inoculation; and their only weak and almost refuted argument is, Are people thus secured, for all their life-time, against the small-pox? Nevertheless, I have the satisfaction to see a partiality for it displayed by the greater part of the public; I have already inoculated many noblemens' children, as well as those of other very respectable inhabitants of Hanover; and, without doubt, I shall always have subjects enough for continuing this inoculation.

On the 9th, 10th, or 11th day, when the inoculated pustule is filled with lymph, and the surrounding inflammation is complete, I am then in the habit of opening it, and to collect the lymph on cotton thread; immediately after, I lay some more cotton thread upon it, which is removed the following day, all of which serves for future inoculations. After this period I have hitherto desisted to repeat collecting more matter from such a pustule, but wish to know how long it might be continued?

I shall esteem it a favour if you will lay these results and questions before Dr. Pearson, and beg he will have the goodness to answer them.

In Langenhagen, a village near Hanover, I have inoculated the clergyman's children, along with four others, which influenced many farmers and peasants to have their own children also inoculated.

From

From Halle, Halberſtadt, and many other places, I have received applications for vaccine matter, which proves that the continent has proportionally as many adherents to the vaccine inoculation as there are in England.

Dr. Ballhorn, an intimate and long acquaintance of mine, has aſſiſted me in moſt of theſe operations, in order that he might viſit the inoculated patients, whenever my time will not admit of it.

In No. 15 and 16 of the Hanoverian Magazine, we have publiſhed our laſt year's inoculations, and we intend continuing the ſame from time to time. With Dr. de Carro, of Vienna, whom Dr. Pearſon knows, and who occupies himſelf greatly with this inoculation, we correſpond.

Only one of our this year's patients has been inoculated for the ſmall-pox; a puſtule was produced upon the inoculated part, accompanied with a ſlight ſurrounding inflammation, but no other effects whatever.

